# *Hepatozoon* species (Adeleorina: Hepatozoidae) of African bufonids, with morphological description and molecular diagnosis of *Hepatozoon ixoxo* sp. nov. parasitising three *Amietophrynus* species (Anura: Bufonidae)

**DOI:** 10.1186/s13071-014-0552-0

**Published:** 2014-12-20

**Authors:** Edward C Netherlands, Courtney A Cook, Nico J Smit

**Affiliations:** Unit for Environmental Sciences and Management, North-West University, Potchefstroom, South Africa

**Keywords:** Adeleorid taxonomy, Apicomplexan phylogenetics, Frog blood haematozoan, Haemogregarine, South Africa, Toad

## Abstract

**Background:**

Haemogregarines comprise a large group of apicomplexan blood parasites. In 1996 all anuran haemogregarines still in the genus *Haemogregarina* Danilewsky, 1885 were reassigned to the genus *Hepatozoon* Miller, 1908. Most (11/15, 73%) African anuran *Hepatozoon* species have been recorded from the family Bufonidae, however, all these are recorded from only two host species, *Amietophrynus mauritanicus* (Schlegel, 1841) and *Amietophrynus regularis* (Reuss, 1833) from Northern and central Africa. To the authors’ knowledge the only description of an anuran haemogregarine from South Africa is *Hepatozoon theileri* (Laveran, 1905), parasitising *Amietia quecketti* (Boulenger, 1895).

**Methods:**

Thin blood smears for morphometrics and whole blood for molecular work, were collected from 32 *Amietophrynus garmani* (Meek, 1897), 12 *Amietophrynus gutturalis* (Power, 1927), and nine *Amietophrynus maculatus* (Hallowell, 1854), in Ndumo Game Reserve and Kwa Nyamazane Conservancy, KwaZulu-Natal, South Africa. Smears were Giemsa-stained, screened for haemogregarines, parasite stages measured, compared to each other and to other described African bufonid haemogregarines. Parasite 18S rDNA was amplified using two apicomplexan-specific primer sets, HepF300/HepR900 and 4558/2733. Resulting sequences of the haemogregarine isolates from the three *Amietophrynus* species were compared with each other and to comparative haemogregarine sequences selected from GenBank.

**Results:**

Morphological characteristics of parasite stages, in particular characteristically capped mature gamont stages, and molecular findings, supported all three haemogregarine isolates from all three *Amietophrynus* species to be the same, a species of *Hepatozoon*, and furthermore different morphologically from other previously recorded bufonid *Hepatozoon* species. The haemogregarine fell within a clade comprising other anuran *Hepatozoon* species and furthermore, within a monophyletic sub-clade along with *H. theileri* and are described as *Hepatozoon ixoxo* sp. nov.

**Conclusions:**

This is the first morphological and molecular account of *Hepatozoon* species within the family Bufonidae from South Africa, a study hoped to encourage the redescription and molecular analysis of those *Hepatozoon* species described in the past from *Amietophrynus* species, as well as to promote the use of both morphological and molecular characteristics in *Hepatozoon* species descriptions. This will aid in comprehensive *Hepatozoon* descriptions, which along with the use of phylogenetic analysis will give a better indication of these parasites possible vectors and life cycle dynamics.

## Background

Haemogregarines comprise a large group of apicomplexan blood parasites recorded from a wide range of tetrapod vertebrates and haematophagous invertebrates [1,2]. Haemogregarines are heteroxenous parasites and the group presently includes three families, namely the Haemogregarinidae Léger, 1911, Hepatozoidae Wenyon, 1926, and Karyolysidae Wenyon, 1926. Within these families there are six genera of blood parasites, differentiated on the sporogonic development in their invertebrate hosts [3,4]. Prior to the clarification of the haemogregarine life cycles in anuran hosts by Desser *et al.* [5], most were placed in the genus *Haemogregarina* Danilewsky, 1885. However in 1996, with further insight into the above, Smith [1] suggested that these haemogregarines were better suited to the genus *Hepatozoon* Miller, 1908 and thus transferred them accordingly. As a result, *Hemolivia* Petit, Landau, Baccam and Lainson, 1990 and *Hepatozoon* are the only two haemogregarine genera with species known to parasitise anuran hosts [2], with the latter currently representing the most common intraerythrocytic protozoan parasites of anurans worldwide [1].

**Figure 1:**
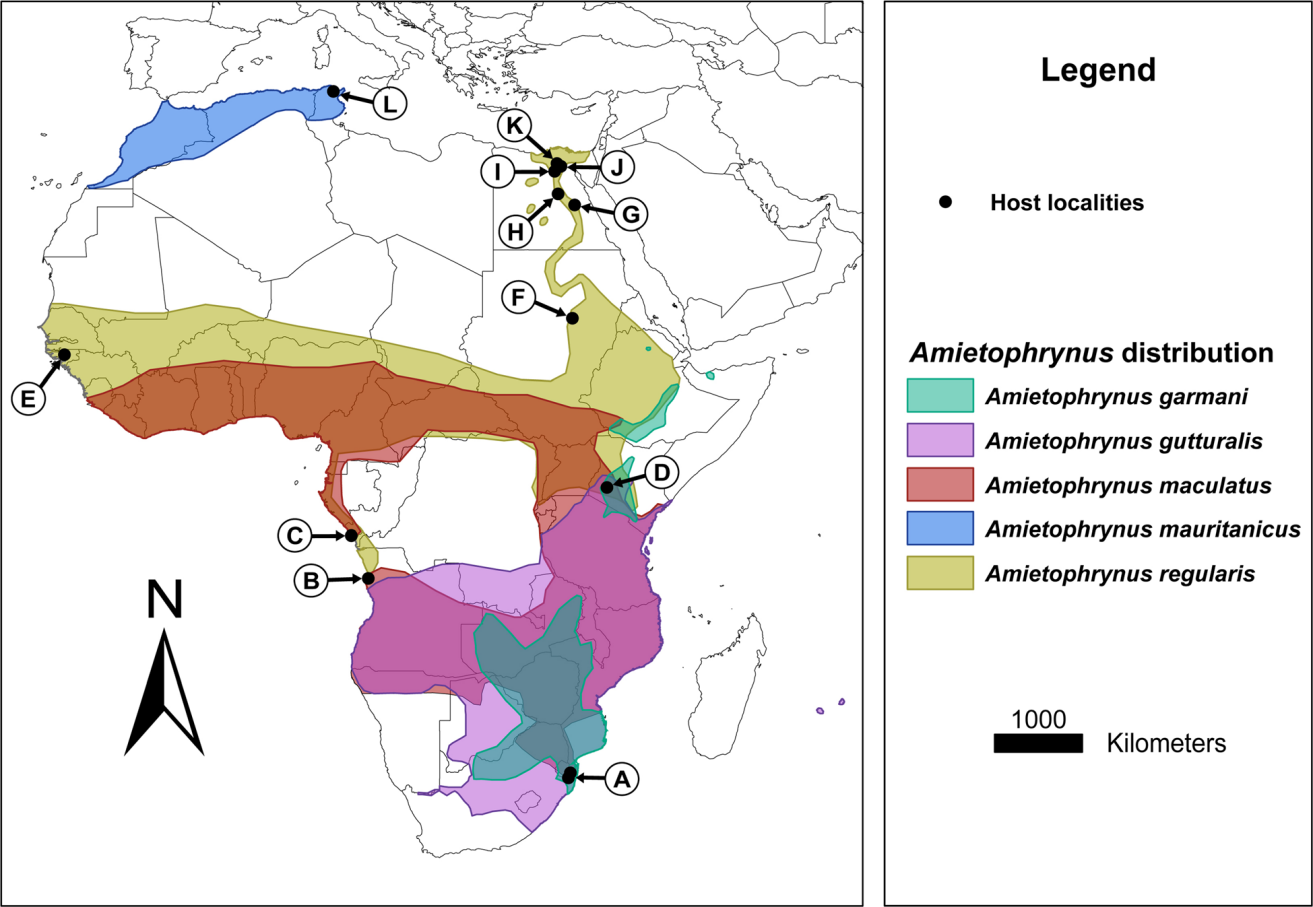
**Map of Africa showing** ***Amietophrynus*** **species distribution pertaining to this study, with locality records of associated** ***Hepatozoon*** **species.** The species *A. garmani*, *A. gutturalis*, *A. maculatus*, *A. mauritanicus* and *A. regularis* (Bufonidae) were found to be parasitised with various *Hepatozoon* species. **A**: Represents the hosts *A. garmani*, *A. gutturalis*, and *A. maculatus* infected with *H. ixoxo* sp. nov. **B**: *A. regularis*, with *H. boueti* and *H. froilanoi*. **C**: *A. regularis*, with *H. lavieri*. **D**: unknown host with *H. moloensis*. **E**: *A. regularis*, with *H. boueti* and *H. pestanae*. **F**: *A. regularis*, with *H. aegyptia*. **G**: *A. regularis*, with *H. magni*. **H**: *A. regularis*, with *H. assiuticus* and *H. francai*. **I**: *A. regularis*, with *H. faiyumensis*. **J**: *A. regularis*, with *H. boueti* and *H. pestanae*. **K**: *A. regularis*, with *H. aegyptia* and *H. boueti*. **L**: *A. mauritanicus*, with *H. tunisiensis*.

According to Netherlands *et al.* [6], the majority (11/15, 73%) of African anuran *Hepatozoon* species have been recorded from the family Bufonidae. Nine of the 11 (81%) species, namely *H. aegyptia* (Mohammed and Mansour, 1963), *H. assiuticus* (Abdel-Rahman, El-Naffar, Sakla and Khalifa, 1978), *H. boueti* (França, 1925), *H. faiyumensis* (Mansour and Mohammed, 1966), *H. francai* (Abdel-Rahman, El-Naffar, Sakla and Khalifa, 1978), *H. froilanoi* (França, 1925), *H. lavieri* (Tuzet and Grjebine, 1957), *H. magni* (Hassan, 1992), and *H. pestanae* (França, 1910) [1], were recorded from the same vertebrate host *Amietophrynus regularis* (Reuss, 1833) in Egypt, Sudan, Nigeria, Guinea-Bissau, the Congo, and from northern Angola [7-16] (see Figure 1). The remaining two species, *H. tunisiensis* (Nicolle, 1904) described from *Amietophrynus mauritanicus* (Schlegel, 1841), and *H. moloensis* (Hoare, 1920) described from an unidentified species (likely *A. regularis*), were reported from Tunisia and Kenya respectively [17,18] (Figure 1) (Table 1). These species descriptions range from 20 to more than 100 years ago and were entirely morphology-based. Furthermore, illustrations and measurements were not standardised and therefore inconsistent, and deposited voucher specimens were not mentioned in any of the descriptions and reports. With so many species described from the same host in largely the same area, the huge diversity of *Hepatozoon* species from the above two hosts may in fact be a false representation of what may truly exist.

**Table 1:** **All described African** ***Hepatozoon*** **species infecting frogs from the family Bufonidae**

***Hepatozoon*** **species**	**Type and other hosts**	**Description mature gamont**	**Locality**	**References**
***Hepatozoon ixoxo*** **sp. nov.**	*Amietophrynus maculatus* (Hallowell, 1854); *Amietophrynus garmani* (Meek, 1897); *Amietophrynus gutturalis* (Power, 1927)	**Mature capped form:** 14.2 ± 0.77 (12.23–16.53) × 7.5 ± 0.51 (6.0–8.5) μm; 3.9 ± 1.20 (2.6–5.9) × 4.0 ± 1.05 (1.4–5.5) μm	NGR and KNC South Africa	Present study
***Hepatozoon aegyptia*** (Mohammed and Mansour, 1963)	*Amietophrynus regularis* (Reuss, 1833) (syn., *Bufo regularis*)	**Mature capped form** ^†^ **:** 14 × 7.3 μm; 3.7 × 2.9 μm	Cairo, Egypt^†^	Mohammed and Mansour [10]†
		Mature capped form*: 13.7 (12.7–14.7) × 8.1 (6.7–8.6) μm; 3.9 (3–4.5) × 3.9 (3.6–4.2) μm	Khartoum, Sudan*	Younis and Saoud [14]*
***Hepatozoon assiuticus*** (Abdel-Rahman, El-Naffar, Sakla and Khalifa, 1978)	*Amietophrynus regularis*	**Mature elongated form** ^†^ **:** 35.4 (30–38) × 4.2 (3.5–5) μm; 4.2 (3–4.7) × 4 (2.8–4.6) μm	Assuit, Egypt^†^	Abdel-Rahman [15]^†^
***Hepatozoon boueti*** (França, 1910) [syn., *Hepatozoon boneti* França, 1925 of Tuzet and Grjebine (1957)]	*Amietophrynus regularis*	**Mature elongated form** ^†^ **:** (16.5–18.5) × 4.5 μm; 4.5 × (3–3.5) μm	Guinea-Bissau^†^	França [7]†
		Mature elongated form*: 22.3 (19–26) × 6 (5–6) μm; 5.2(4–6) × 4.2 (3–6) μm	Cairo and Giza, Egypt*	Mohammed and Mansour [11]*
***Hepatozoon faiyumensis*** (Mansour and Mohammed, 1966)	*Amietophrynus regularis*	**Mature elongated form** ^†^ **:** 15.5 (13–17) × 4.5 (4–5) μm; 4.5 (3–5) × 3.9 (3–5) μm	Faiyum, Egypt^†^	Mansour and Mohammed [11]^†^
***Hepatozoon francai*** (Abdel-Rahman, El-Naffar, Sakla and khalifa, 1978)	*Amietophrynus regularis*	**Mature capped form** ^†^ **:** (18.5–20) × (2.7–3) μm	Assuit, Egypt^†^	Abdel-Rahman et al. [15]^†^
***Hepatozoon froilanoi*** (França, 1925)	*Amietophrynus regularis*	**Mature elongated form** ^†^ **:** (16.5–21) × 4.5 μm; (6–7.5) × 4.5 μm	Luanda, Angola^†^	França [8]^†^
***Hepatozoon lavieri*** (Tuzet and Grjebine, 1957)	*Amietophrynus regularis*	**Possible gamont form** ^†^ **:** 20 μm in length	Pointe-Noire, Congo^†^	Tuzet and Grjebine [9]^†^
***Hepatozoon magni*** (Hassan, 1992)	*Amietophrynus regularis*	**Capped form**†**:** 15.05 (14.2–16.8) × 7.7 (6.3–9.8) μm; 3.4 (2.8–4.2) × 3.4 (2.8–4.2) μm	Qena, Egypt^†^	Hassan [16]^†^
		**Mature elongated form** ^†^ **:** 22.5 (21.2–26.6) × 8.2 (7.7–9.1) μm; 4.3 (4.1–4.6) × 4.5 (4.3–4.8) μm		
***Hepatozoon moloensis*** (Hoare,1920)	*Amietophrynus spp.*	**Mature capped form** ^†^ **:** 18.8 × 7.8 μm	Molo, Kenya^†^	Hoare [18]^†^
***Hepatozoon pestanae*** (França, 1910)	*Amietophrynus regularis*	**Mature capped form** ^†^ **:** 12 × 4.5 μm; 3.7 μm	Guinea-Bissau^†^	França [7]†
		Mature capped form*: 13 (12–17) × 5 (4.5–5) μm; 4.2 × 3.7 μm	Giza, Egypt*	Mohammed and Mansour [13]*
***Hepatozoon tunisiensis*** (Nicolle, 1904)	*Amietophrynus mauritanicus* (Schlegel, 1841) [syn., Bufo *mauritanicus*]	**Mature capped form** ^†^ **:** (12–15) × 8 μm	Tunis, Tunisia^†^	Nicolle [17]^†^

^†^original species description, * redescription or other descriptions of the same species. NGR: Ndumo Game Reserve; KNC: Kwa Nyamazane Conservancy.

Measurements given where possible, length mean ± standard deviation (range) × width mean ± standard deviation (range); nucleus length mean ± standard deviation (range) × nucleus width mean ± standard deviation (range).

The aim of this study was therefor to elucidate, via traditional morphological description of peripheral blood stages and molecular techniques, the identity of the *Hepatozoon* species found to infect three *Amietophrynus* species from South Africa and to determine whether they represent a single species or three cryptic species, such as what has been recorded, solely morphologically, from other *Amietophrynus* species further north in Africa. According to the authors’ knowledge the only record of an anuran haemogregarine from South Africa is *Hepatozoon theileri* (Laveran, 1905), described from the host *Amietia quecketti* (Boulenger, 1895), family Pyxicephalidae [6]. This paper therefore presents the first morphological and molecular account of a *Hepatozoon* species parasitising members of the Bufonidae from South Africa, and establishes the phylogenetic basis for all other bufonid *Hepatozoon* species in Africa.

## Methods

Map (Figure 1) was created through ArcGIS 10.1 [19] using spatial data downloaded from IUCN Red List of Threatened Species [20].

### Frog collection and husbandry

Specimens of *Amietophrynus garmani* (Meek, 1897), *Amietophrynus gutturalis* (Power, 1927), and *Amietophrynus maculatus* (Hallowell, 1854) [21,22], were collected by hand at night in the Ndumo Game Reserve, North Eastern KwaZulu-Natal (KZN), South Africa, from a total of eight sites. These include three temporary pans (26°51′54.5″S, 32°09′59.9″E; 26°53′51.6″S, 32°12′57.2″E; and 26°52′53.5″S, 32°15′03.4″E), one wetland (26°54′08.2″S, 32°14′15.0″E), two riverine (26°54′18.5″S, 32°19′24.7″E; and 26°52′57.8″S, 32°18′41.8″E), one lake (26°53′35.6″S, 32°17′45.2″E), and one man-made pond at the campsite (26°54′33.8″S; 32°18′50.5″E) (and therefore anthropogenically impacted); as well as from the Kwa Nyamazane Conservancy from a total of three sites, including two temporary pans (27°23′43.9″S, 32°08′33.7″E and 27°24′35.1″S, 32°08′47.8″E), and one riverine site (27°23′26.5″S, 32°08′24.2″E). All these sites in KZN were visited during the warmer months of February and November 2012, April and November 2013 and February and April 2014. Two specimens, both *A. maculatus*, collected (26°54′18.5″S, 32°19′24.7″E) in April 2013, and found to be heavily parasitised with *Hepatozoon* species (3.8% and 9.2% respectively), were kept, maintained in vivarium and fed on common garden crickets (*Gryllus bimaculatus*) for over a period of a year to monitor peripheral blood parasite stages and parasitemia on a bimonthly basis.

### Frog blood smear preparation and screening

Blood was taken from the femoral arteries or veins and thin blood smears prepared, air-dried, fixed and stained using Giemsa-stain (FLUKA, Sigma-Aldrich, Steinheim, Germany). Subsequently smears were screened at 100×, images captured and parasites measured as described previously [6]. Parasitaemia was calculated per 100 erythrocytes, with ~10^4^ erythrocytes examined per blood smear, following previous methods [23]. Descriptive statistics of the length (L) and width (W) results of mature gamont stages were compared between the three frog species using one way ANOVA (IBM SPSS V22). The remaining blood, that was not used in blood smear preparation, was placed in sterile 0.5 ml reaction tubes with an equal volume of 70% molecular grade ethanol to be processed molecularly at a future date. This study received the relevant ethical approval (North-West University ethics approval no: NWU-00005-14-S3).

### DNA extraction and phylogenetic analysis

Peripheral blood, obtained from parasitised specimens of two *A. garmani*, two *A. gutturalis*, and four *A. maculatus* was transferred to sterile 0.5 ml reaction tubes. Additionally the blood of one highly parasitised *Amietia quecketti* with *Hepatozoon theileri* from a previous study [6], was also used in order to obtain a longer sequence for comparison. DNA was extracted from the samples using the standard protocol for human or animal tissue and cultured cells as detailed in the NucleoSpin®Tissue Genomic DNA Tissue Kit (Macherey-Nagel, Düren, Germany). To amplify apicomplexan parasite 18S rDNA from the total DNA extracted, polymerase chain reaction (PCR) sequence runs were undertaken in a Bio-Rad C1000 Touch™ Thermal Cycler (Bio-Rad, Hemel Hempstead, UK). PCR reactions were performed in volumes of 25 μl, using 12.5 μl Thermo Scientific PCR master mix (2X) (0.05 U/μl Taq DNA Polymerase reaction buffer, 0.4 mM of each dNTP, and 4 mM MgCl2), 1.25 μl of each primer, and at least 25 ng of DNA. The final reactions volume was made up of PCR grade nuclease free water (Thermo Scientific). Identification of *Hepatozoon* species were initially completed using the *Hepatozoon* specific SIGMA primer set HepF300: 5′-GTTTCTGACCTATCAGCTTTCGACG-3′ and HepR900 5′-C AAATCTAAGAATTTCACCTCTGAC-3′. The PCR reactions were run targeting a fragment (approximately 600 bp) of the 18S rDNA gene [24]. Conditions for PCR are detailed according to previous methods [6]. A second PCR was carried out using another apicomplexan-specific parasite SIGMA primer set 4558: 5′-GCTAATACATGAGCAAAATCTCAA-3′ and 2733: 5′-CGGAATTAACCAGACAAAT-3′ [25], targeting a longer fragment (approximately 1,120 bp) of the 18S rDNA gene for all samples found positive with *Hepatozoon* species. PCR conditions were as follows: initial denaturation at 94°C for 3 min, followed by 40 cycles, entailing a 94°C denaturation for 1 min, annealing at 55°C for 2 min with an end extension at 72°C for 2 min, and following the cycles a final extension of 72°C for 10 min [25]. Resulting amplicons were visualised under UV on a 1% agarose gel stained with gel red using a Bio-Rad GelDoc Imaging System (Bio-Rad, Hemel Hempstead, UK). PCR products were sent to a commercial sequencing company (Inqaba Biotechnical Industries (Pty) Ltd. Pretoria, South Africa) for purification and sequencing in both directions.

From the resulting sequences chromatogram-based contigs were generated and trimmed using Geneious Ver. 7.1 [26], for further analysis. All the sequences have been uploaded onto GenBank under the accession numbers [GenBank: KP119770 – KP119773].

Sequences were identified as those of *Hepatozoon* using the Basic Local Alignment Search Tool (BLAST) [27], and comparative haemogregarine sequences were selected for further phylogenetic analysis. In addition to the four sequences obtained from the three *Amietophrynus* species and one *A. quecketti* in this study, 18 additional sequences were acquired from GenBank, 15 *Hepatozoon* as well as a single *Hemolivia* [GenBank: KC512766], *Haemogregarina* [GenBank: KF257925] and *Dactylosoma* [GenBank: HQ224957] (used as an outgroup) species. All phylogenetic analyses were undertaken using the Geneious (Ver. 7.1) bioinformatics software package. Sequences were aligned using the MUSCLE algorithm [28], for both Maximum likelihood (ML) and Bayesian inference (BI) analyses. The alignment consisted of 22 taxa and 690 bp long. ML phylogeny was completed using PhyML Ver. 2.4.4 plugin [29], according to the estimated AIC criterion using jModelTest Ver. 2.1.5 [30]. The chosen parameters of the substitution model were TVM + G. Nodal support was undertaken with 1000 Bootstrap replicates. Tree topology search was completed using Nearest Neighbour Interchange (NNI) and Subtree Pruning and Regrafting (SPR). BI phylogeny was implemented using MrBayes Ver. 3.2.1 plugin [31], under the estimated parameters as part of the analysis. The analysis was run twice for 2×10^6^ generations; saving one tree every 1000 generations; with burn-in values of 100,000 and four chains at a temperature of 0.20. The log-likelihood values of the sample point were plotted against the generation time and all the trees prior to reaching stationary were discarded, no burn-in samples were retained. Remaining trees were combined in a 50% majority consensus tree, in which frequency of any particular clade represents the posterior probability [31]. Strict consensus trees for both ML and BI trees were generated and visualised using Tree-view [4].

## Results

### General observations

Three species from the family Bufonidae were sampled from the Ndumo Game Reserve and the Kwa Nyamazane Conservancy, KZN (*A. garmani* and *A. maculatus*, and *A. gutturalis* respectively). Of the 53 individuals collected 16 (30.1%) were found to be infected with haemogregarines; from the Ndumo Game Reserve, 1/23 (4.3%) from *A. garmani* and 6/9 (66.7%) from *A. maculatus*; from the Kwa Nyamazane Conservancy, 5/9 (55.6%) from *A. garmani* and 4/12 (33%) from *A. gutturalis*. These infections were recorded from frogs inhabiting mainly riverine sites with the exception of two individuals from two separate temporary pans in the Kwa Nyamazane Conservancy. It was found that frogs collected from other sites such as the wetland, lake and campsite were not infected with the parasite. Five different stages were found in the peripheral blood. Trophozoite, meront and merozoite stages were rare and were only observed within the blood smears of two individuals of *A. maculatus* collected in April 2013 from the Ndumo Game Reserve. The most frequently encountered stages were immature and mature gamont stages, the latter stage being the most abundant across all the parasitised specimens. The mature gamont was characterised by a well-developed cap, typically staining pink, situated at one pole of a delicate capsule. According to the morphological measurements (Table 2) and other morphological features (as described in the detailed description) of the gamont stages, it is suggested that the *Hepatozoon* species found parasitizing all three toads is one of the same. This was concluded in spite of the size of the mature gamonts measured from *A. garmani* being smaller, with statistical differences considered to be significant at p < 0.05 compared to both *A. gutturalis* and *A. maculatus* p = 0.000. The latter two appeared to be very similar p = 0.628, with no significant difference observed. Pending the molecular outcome, this was attributed to intra-species variation. Throughout the collection of the host specimens no vectors were observed feeding on the frogs.

**Table 2:** **Morphometrics of** ***Hepatozoon ixoxo*** **sp. nov. from the** ***Amietophrynus*** **species collected in this study**

**Frog hosts by species**	**Measurements of the different stages found in the different host species**		
	**Trophozoites:**	**Immature gamonts:**	**Mature gamonts:**
*Amietophrynus garmani* (Meek, 1897)		13.2 ± 0.6 (11.6–14.0) × 5.7 ± 0.3 (5.0–6.2) μm;	13.8 ± 0.7 (12.2–15.5) × 7.4 ± 0.6 (6.0–8.5) μm;
		5.0 ± 0.7 (3.6–6.2) × 4.1 ± 0.6 (2.6–5.1) μm (n = 16)	3.8 ± 1.3 (2.7–5.1) × 3.5 ± 1.2 (1.4–4.7) μm (n = 66)
*Amietophrynus gutturalis* (Power, 1927)		14.7 ± 0.5 (13.7–16.0) × 6.7 ± 0.2 (6.1–7.1) μm;	14.7 ± 0.9 (12.3–16.5) × 7.6 ± 0.3 (7.1–8.2) μm;
		4.9 ± 0.7 (3.7–6.9) × 3.90 ± 0.9 (2.0–6.1) μm (n = 26)	4.1 ± 1.1 (3.2–5.3) × 4.0 ± 1.1 (2.5–4.8) μm (n = 20)
*Amietophrynus maculatus* (Hallowell, 1854)	9.5 ± 1.3(6.9–11.7) × 3.3 ± 0.5 (2.2–4.8) μm (n = 23)	14.4 ± 0.4 (13.6–15.3) × 6.1 ± 0.5 (4.4–6.8) μm;	14.5 ± 0.5 (13.4–15.7) × 7.6 ± 0.4 (6.6–8.5) μm;
		5.2 ± 0.6 (4.3–6.3) × 4.2 ± 0.5 (2.7–4.9) μm (n = 26)	3.9 ± 0.5 (3.0–5.1) × 4.3 ± 0.5 (2.7–4.9) μm (n = 46)
Total average		14.2 ± 0.7 (11.6–16.0) × 6.4 ± 0.6 (4.4–7.9) μm;	14.2 ± 0.77 (12.23–16.53) × 7.5 ± 0.51 (6.0–8.5) μm;
		5.0 ± 1.4 (3.3–7.3) × 3.9 ± 0.8 (1.9–6.1) μm (n = 102)	3.9 ± 1.20 (2.6–5.9) × 4.0 ± 1.05 (1.4–5.5) μm (n = 132)

Measurements given in μm, length mean ± standard deviation (range) × width mean ± standard deviation (range); nucleus length mean ± standard deviation (range) × nucleus width mean ± standard deviation (range) (n = number measured).

### Taxonomic summary

Phylum Apicomplexa Levine, 1970.

Family Hepatozoidae Wenyon, 1926.

Genus *Hepatozoon* Miller, 1908.

Description of *Hepatozoon ixoxo* sp. nov. Netherlands, Cook, and Smit, 2014.

#### Morphology

**Figure 2:**
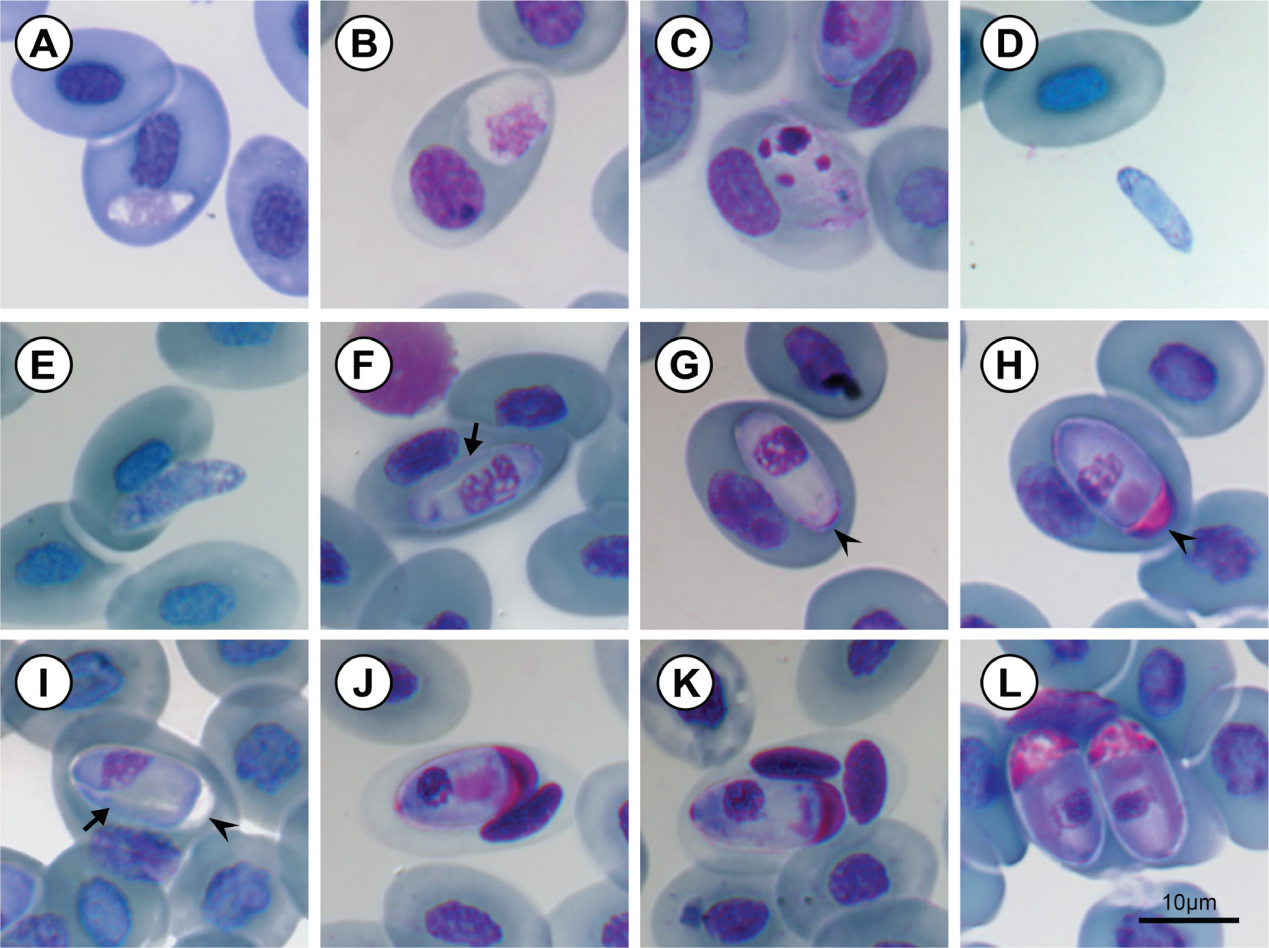
***Hepatozoon ixoxo*** **sp. nov. (A-L) in the peripheral blood of the frog** ***Amietophrynus maculatus*** **Hallowell, 1854 (Bufonidae). A**: Trophozoite. **B**: Possible pre-meront. **C**: Meront stage. **D**: Extracellular or free merozoite stage. **E**: Merozoite, showing likely entry into an erythrocyte. **F**: Immature gamont displaying a recurved tail (arrow) and loosely arranged nucleus. **G**: Immature gamont before development of the capsular cap at the truncate pole (arrow-head). **H-L**: Mature gamonts, note the well-developed capsular cap forming at the truncate pole (arrow-head), staining pink (**H**, and **J-L**), with a prominently visible compact nucleus. **I**: Mature gamont displaying a recurved tail (arrow), note the cap did not stain pink. **J**: Dehaemoglobinisation of host cell, often followed by, lysing of the host cell nucleus (**K)**. **L**: Double infection of a single erythrocyte. All images captured from the hapantotype slide (NMB P 368). Scale bars: 10μm.

**Trophozoite:** irregular to ovoid shape, often vacuolated cytoplasm, measuring 9.5 ± 1.3 (6.9–11.7) long by 3.3 ± 0.7 μm (2.2–4.8) wide (n = 23), nucleus with loose chromatin, staining pink to purple, measuring 3.4 ± 0.8 μm (2–4.8) long by 2.2 ± 0.5 μm (1.5–43.8) wide (Figure 2A).

**Meront:** globular in shape, with a foamy cytoplasm, staining white, measuring 8.2 ± 1.3 μm (7–9.5) long by 8.5 ± 2 μm (7.1–9.9) wide (n = 3). The nucleus has loosely arranged chromatin, extending outwards, staining light pink, measuring 4.6 ± 1.6 μm (3.2–6.3) long by 5.1 ± 1.4 μm (3.5–5.9) wide (n = 3) (Figure 2B, C). Possible later-meront stage, with granulated cytoplasm, staining light purple, with four distinct and condensed nuclei, staining dark purple (Figure 1C).

**Merozoite:** elongated in shape, tapering toward one pole (possibly anterior), rounded at the other (possibly posterior), found either free in between the erythrocytes (Figure 2D), or possibly entering or leaving an erythrocyte, vacuolated cytoplasm, staining light blue, measuring 12.9 ± 1.9 μm (10.7–14.4) long by 3.2 ± 0.8 μm (2.5–4) wide, nucleus staining similarly light blue with a condensed appearance, measuring 4.7 ± 0.6 μm (4–5.2) long by 3.1 ± 0.8 μm (2.5–4) wide (n = 3).

**Immature gamonts:** oval shaped without cap or cavity at truncate pole (Figure 2F-G arrow-head), cytoplasm staining whitish-blue or purple, measuring 14.2 ± 0.7 μm (11.6–16) long by 6.4 ± 0.6 μm (4.4–7.9) wide (n = 102). Irregular oval shaped nucleus, with loose chromatin, staining dark blue or purple, measuring 5 ± 1.4 μm (3.3–7.3) long by 3.9 ± 0.8 μm (1.9–6) wide. Infrequently a recurved tail was observed (Figure 2F arrow), the nucleus lying nearer to the anterior pole (the broader pole of the parasite) (Figure 2F).

**Mature gamonts:** oval shaped with a well-developed cap/cavity at the truncate pole (Figure 2H-I arrow-head) or folded region of the parasite, often staining pink (Figure 2H-L); seemingly encased by a thick parasitophorous vacuole or delicate capsule, gamont with cap measuring 14.2 ± 0.8 μm (12.2–16.5) long by 7.5 ± 0.5 μm (6–8.5) wide (n = 133); excluding cap measuring 11.8 ± 2.4 μm (8–14.3) long, width remaining the same. A small recurved tail seldom visible (Figure 2I arrow). Whitish-blue to purple staining cytoplasm, with an irregular, oval-shaped, blue or dark-purple staining nucleus measuring 3.90 ± 1.20 μm (2.6–5.9) long by 4 ± 1.1 μm (1.5–5.5) wide; in some cases the gamont would cause dehaemoglobinisation of the host cell (Figure 2J-K), as well as lysing of its nucleus (Figure 2K).

#### Type host

*Amietophrynus maculatus* (Hallowell, 1854), Anura: Bufonidae.

#### Other hosts

*Amietophrynus garmani* (Meek, 1897), and *Amietophrynus gutturalis* (Power, 1927), Anura: Bufonidae.

#### Vector

Unknown.

#### Type locality

The specimens were collected along the Phongolo River (26°54′18.5″S, 32°19′24.7″E) in the Ndumo Game Reserve, KwaZulu-Natal, South Africa.

#### Type material

Hapantotype, 1× blood smear from *Amietophrynus maculatus* NMB P 368; parahapantotypes, 1× blood smear from *Amietophrynus garmani* NMB P 369 and *Amietophrynus gutturalis* NMB P 370 respectively, deposited in the Protozoan collection of the National Museum, Bloemfontein, South Africa.

#### General

In accordance with section 8.5 of the ICZN’s International Code of Zoological Nomenclature, details of the new species have been submitted to ZooBank with the life science identifier (LSID) zoobank.org:pub: 3DA4C637-3508-4EC2-A7BC-253EDCD07CB1.

#### Gene sequences

The 18S ribosomal RNA gene sequences have been uploaded onto GenBank under the accession numbers [GenBank: KP119770 – KP119772].

#### Etymology

The species is named after the Zulu name for frog or toad, since the hosts consist of various frog species and the parasite species type locality is in KwaZulu-Natal. “*Ixoxo*” is correctly pronounced [i:ǁT:ǁo] in which each 'x' is a single lateral click that is best approximated in Indo-European languages with a 'ch'. “*Ixoxo*” is pronounced “ee-ch-o-ch-o”.

#### Molecular analysis

**Figure 3:**
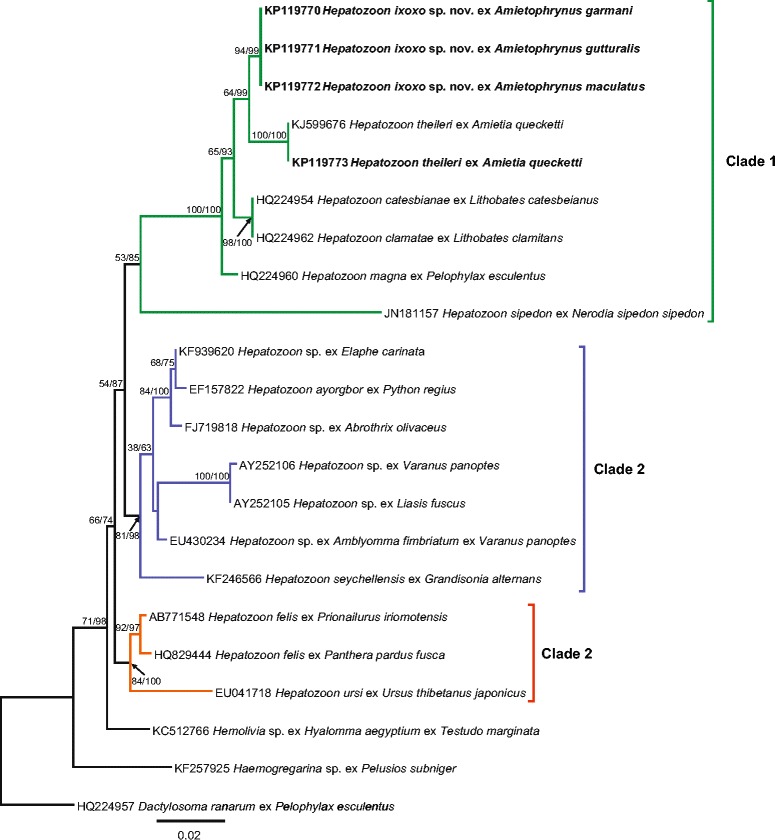
**Phylogenetic position of** ***Hepatozoon ixoxo*** **sp. nov., based on 18S rDNA gene sequences.** Tree topology was identical across Maximum Likelihood (ML), and Bayesian inference (BI). Therefore the nodal support values for each analysis (ML/BI) are represented on the ML tree. ML tree was constructed using PhyML under the parameters of the substitution model being TVM + G with 1000 Bootstrap replicates, the BI tree was constructed using MrBayes under the estimated parameters as part of the analysis. New hapanotypes are represented in bold. Distinct clades are represented in different colours (green/blue/orange).

Once edited for phylogenetic analysis, four high-quality sequences of 1033 base pairs (bp), one from each of the three *Amietophrynus* species and one from an *A. quecketti* infected with *H. theileri*, were produced using 4558 and 2733 primer sets, targeting part of the 18S rDNA gene [25]. The overall topology of the generated ML and BI phylogenetic trees were identical and nodal support of each analysis is represented on the ML tree as ML/BI (Figure 3). The phylogenetic tree comprises three distinct clades. The haemogregarine isolates of all three *Amietophrynus* species, as well as that of the *A. quecketti*, were found to fall within the first clade comprising mostly anuran *Hepatozoon* species. Furthermore, that from *A. quecketti* was found to be, as expected, identical to a previously deposited isolate of *H. theileri* [6]. The three isolates [GenBank: KP119770 – KP119772] from the *Amietophrynus* species were all found to be identical and to form a sister taxon to *H. theileri* [GenBank: KJ599676; KP119773] (see Figure 3).

### Bimonthly peripheral blood observations

Two specimens of highly parasitised *A. maculatus* (collected in April 2013) were kept in a vivarium and their blood screened for peripheral blood stages on a bimonthly basis, from May 2013 to and including May 2014. This was done in order to monitor their parasitaemia levels in the absence of a vector, as well as to observe any changes in the peripheral blood stages. The parasitaemia over a period of one year for the first specimen averaged 4.8% (3.6‒6.6%). May 2014 had the lowest parasitemia of 3.6% and July 2013 the highest of 6.6%. The second specimen’s parasitemia averaged 12.5% (8.2‒19.2%). May 2014 had the lowest parasitemia of 8.2% and July 2013 the highest of 19.2%. In both specimens, upon first collection (April 2013), trophozoite, merozoite, immature and mature gamont stages were observed. As the observation period progressed from there on, only immature and mature gamont stages were observed, mature gamont stages being the predominant stage. Parasitaemia levels peaked within the first four months (July 2013) and then steadily decreased over the next nine months to a similar parasitaemia level as was first recorded in May 2013. No extracellular or extracapsular gamonts were ever observed during this period in the peripheral blood.

## Discussion

Morphological characteristics of peripheral blood parasite stages strongly suggested that all three *Amietophrynus* species were infected with the same species of haemogregarine, likely, based on the lack of peripheral division stages and the recommendations on anuran haemogregarines of Smith [1], a species of *Hepatozoon*. This was supported molecularly, with sequences of haemogregarine isolates from all three frog species being identical and falling within a clade comprising other anuran *Hepatozoon* species (see Figure 3).

The capped mature gamont form of the *Hepatozoon* described here was the most frequently encountered stage throughout the current study, as well as in several other studies of bufonid *Hepatozoon*, and is thus consequently considered further within the following discussion. In terms of morphometrics the capped mature gamonts of *H. ixoxo* sp. nov. are most similar to the capped forms of *H. aegyptia*, *H. magni* and *H. tunisiensis* (see Table 1). Even though the present study’s capped gamont form shares a number of morphological similarities to all three of the above described parasites, it does not conform entirely to any of these three. All four parasites, including this study’s *Hepatozoon*, are characterised by being tightly enclosed in a delicate capsule that does not appear to interfere with the staining of the parasite as do those of chelonians [23,32,33], and all show some form of a cap-like structure or space at one or both poles that is filled with a dark-staining intracapsular material (Figure 1H-L). In addition, all four species’ gamonts appear to be folded-over on themselves forming two branches within the capsule, the nucleus of all four situated within the wider of the two branches. Of the four similar species, *H. tunisiensis* does not have a definite cap at the truncate end (the region of the fold) (Figure 1G-I, arrow-head) of the parasite, the intracapsular material scattered frequently around the gamont and accumulating towards the poles of the encapsulated parasite, forming, in comparison to the other three, weak cap-like structures. Similarly, the parasite described in this study shows some scattered intracapsular material (Figure 1H, J, K), but unlike *H. tunisiensis* it has a distinct cap similar to those of *H. aegyptia* and *H. magni*. Furthermore, Nicolle [19] described *H. tunisiensis* to cause no hypertrophy of the host cell, this being equally true of *H. magni* described by Hassan [16], which is in direct contrast to *H. aegyptia* and *H. ixoxo* sp. nov., which both cause fragmentation of the host cell nucleus as well as dehaemoglobinisation of the host cell cytoplasm (see Figure 1J-K). Even though it would appear that *H. ixoxo* sp. nov. conforms closely both in size and morphological characteristics to *H. aegyptia*, during the period of a year in which peripheral blood stages were monitored bimonthly, no elongated forms, such as those of *H. aegyptia* and *H. magni*, were ever observed for *H. ixoxo* sp. nov.

Geographically, all three frog species examined in this study are sympatric. The finding that the haemogregarine isolates from these three frog species represents the same *Hepatozoon* species, *H. ixoxo* sp. nov., is thus not surprising. In addition, even though *A. garmani*, *A. gutturalis* and *A. maculatus* are sympatric to *A. regularis*, the closest geographical overlap to the present study’s site is Kenya, which even though *A. regularis* has been examined there in the past no haemogregarine infections were ever reported [34]. Since the above frog species are found to occur sympatrically in Kenya, it would be expected that the same is true for their parasites, *H. ixoxo* sp. nov. and *H. aegyptia* respectively, as can be seen in the case of *H. catesbianae* and *H. clamatae* which along with their hosts occur sympatrically across Nova Scotia [35]. It may be that the *Hepatozoon* species of African bufonids are locality specific as are some of those infecting African chelonians [23,33,36], or the snakes of Florida in the U.S.A. [37]. As mentioned previously, *H. ixoxo* sp. nov., was found parasitising frogs inhabiting temporary pan and riverine sites as compared to no visible infections in frog hosts inhabiting wetland, lake and campsites. It may suggest that the infection is especially dependent on a vector that may be limited to a specific habitat. Hence, considering the above morphological and geographical aspects, it is strongly suggested that *H. ixoxo* sp. nov. is a new species.

Molecularly, as mentioned above, *H. ixoxo* sp. nov. fell within the first clade (represented in green) comprising anuran *Hepatozoon* species (Figure 3). The second clade (represented in blue) comprises mainly of reptile hosts with the exception of a *Hepatozoon* sp. [GenBank: FJ719818] from the rodent host *Abrothrix olivaceus* (Waterhouse, 1837) and surprisingly *Hepatozoon seychellensis* Harris, Damas-Moreira, Maia et Perera 2014 [GenBank: KF246566], from the caecilian host *Grandisonia alternans* (Stejneger, 1893) [38]. The third clade (represented in orange) comprises *Hepatozoon* spp. from larger mammal hosts and falls outside other *Hepatozoon* spp. from amphibian and reptile hosts.

The first clade, dominated by anuran *Hepatozoon*, comprises one exception, that of *Hepatozoon sipedon* Smith, Desser et Martin, 1994 [GenBank: JN181157], which is a *Hepatozoon* species of both an anuran, *Lithobates pipiens,* as well as a snake, *Nerodia sipedon,* vertebrate host, thus forming a sister taxon on its own within the larger first clade (represented in green). Such a result is not unanticipated as it is a well-known described three-host (snake, frog and mosquito *Culex pipiens* and *Culex territans*) life cycle [6,39]. Since *H. ixoxo* sp. nov. falls along with *H. theileri*, it may suggest that *H. ixoxo* sp. nov. follows a two-host life cycle such as that suggested for *H. theileri* in the latter’s redescription by Netherlands et al. [6]. Furthermore, this small clade comprising *H. ixoxo* sp. nov. and *H. theileri* falls within the same monophyletic sub-clade containing *H. catesbianae*, *H. clamatae* and *H. magna. Hepatozoon catesbianae* and *H. clamatae*, and presumably *H. magna* [4] follow a two-host life-cycle which does not include a cystic stage within the intermediate vertebrate host and can be experimentally transmitted to culicine mosquito definitive hosts which subsequently demonstrate sporogonic stages as described by several authors [4,5,39,40] (see Figure 3). Once again, the positions of the above *Hepatozoon* species including that of *H. sipedon*, supports the theory of Barta et al. [4] on the co-evolution of haemogregarines and their definitive hosts. Overall, the tree indicates with the phylogenetic placement of larger mammal hosts (clade 3), small rodent and snake hosts (clade 2) and frog, snake associations (clade 1), the potential of *Hepatozoon* host, prey and vector interactions.

## Conclusions

Future research should include the identification of possible definitive hosts or vectors such as mosquitoes as well as experimental transmission studies. Considering the close morphological resemblance of all four species of *Hepatozoon*, the present study’s *H. ixoxo* sp. nov. with *H. aegyptia*, *H. magni* and *H. tunisiensis*, it would be particularly beneficial to the study of *Hepatozoon* species of the Bufonidae if the latter three species could be isolated once more for molecular analysis. For instance, it can be seen that there is a small degree of intraspecies variation with regards to the size of the capped mature gamont of *H. ixoxo* sp. nov. between the three host species. The size of the gamont measured from *A. garmani* is significantly smaller than the gamonts from both *A. gutturalis* and *A. maculatus*. It may have been suggested in the past then, from a solely morphological point, that the *Hepatozoon* from *A. garmani* may be a different species. If intraspecific variation of sporogonic stages of a single species of *Hepatozoon* can occur between different vector individuals of the same host species [41], it is possible that this could also be true of the peripheral blood stages. The findings of this study strongly advocate the molecular analysis of those *Hepatozoon* species described in the past from *A. regularis*. This, it is believed will aid in determining with more accuracy whether or not they are all of the same species or if they are in fact different species, along with providing a better indication, with the use of their phylogenetic placement, of their possible vectors and life cycle dynamics.
